# Primary intramedullary spinal cord lymphoma misdiagnosed as longitudinally extensive transverse myelitis: a case report and literature review

**DOI:** 10.1186/s12883-023-03383-4

**Published:** 2023-10-04

**Authors:** Huizhen Ge, Li Xu, Huajie Gao, Suqiong Ji

**Affiliations:** Department of Neurology, Tongji Hospital, Tongji Medical College, Huazhong University of Science and Technology, Wuhan, 430030 China

**Keywords:** Primary intramedullary spinal cord lymphoma, Longitudinally extensive transverse myelitis, Misdiagnosis

## Abstract

**Background:**

Primary intramedullary spinal cord lymphoma (PISCL) is rare and easily misdiagnosed with the lack of typical clinical features and non-specific imaging manifestations.

**Case presentation:**

A 49-year-old man was admitted to our hospital because of persistent limbs numbness, pinprick-like pain in the posterior neck and unsteady gaits. He has brisk tendon reflexes and positive Babinski’s sign. Magnetic resonance imaging (MRI) of the cervical spine showed an abnormal signal with aberrant reinforcement at medulla oblongata and the level of C1-C7. He was clinically diagnosed as longitudinally extensive transverse myelitis (antibody-negative). Steroid pulse therapy was administered and resulted in reduced symptoms. One month later, his situation was exacerbated compared to the onset. We launched a new cascade of steroid pulse therapy. But it did not improve his symptoms. Finally, the biopsy pathology confirmed PISCL. Chemotherapy, radiotherapy and zanubrutinib were administered and until now about 3 years into treatment the patient is still survival.

**Conclusions:**

Based on our case and literature review, we recommend that spinal onset patients react ineffectively to standard immunoglobulins or hormonal treatments or experience a relapse after a short time relief should take PISCL into consideration.

## Background

Primary central nervous system lymphoma (PCNSL) is rare, comprising less than 1% of all lymphoma [1], which is confined to the eyes, brain, leptomeninges and spinal cord with no evidence of systemic disease [2]. Primary spinal intramedullary lymphoma is a rare form, comprising less than 1% of all PCNSL [3]. But studies have shown that number of patients is increasing regardless of immunologic inadequacy or not [4]. The majority of patients have a later onset in 50, mainly involving the thoracic and cervical segment with poor long-term survival and high mortality. Despite some cases previously reported, the characteristics and progress of the disease still remains poorly understood along with delayed diagnosis and hence delayed in treatment [5–7]. Therefore it should take this disease into consideration when the patient has myelopathy with unknown etiology. Herein, we report a rare case that initially was diagnosed as longitudinally extensive transverse myelitis and finally confirmed as PISCL.

## Case presentation

**Fig. 1:**
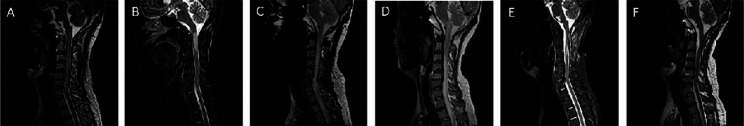
Serial sagittal MR images at presentation and follow-up. Initial sagittal image **(A)** revealed patchy hyperintense signal change of the medulla oblongata and cervical spinal cord. Repeated MRI after 0.5 months **(B)** * showed the lesion reduced. MRI at 1.5 months from presentation **(C)**^Δ^ showed apparent enlargement of the lesion. MRI at 2 months from presentation **(D)**^#^ showed similar lesion. MRI at 5 months from presentation (E)^**^ revealed the lesion significantly reduced. Further reduced lesion was shown in MRI at 6 months from presentation **(F)**. ^*^ Steroid pulse therapy was completed before this MRI. ^Δ^ A relapse. ^#^ Another steroid pulse therapy was completed before this MRI. ^**^ Combination chemotherapy regimens had been proceeding

**Fig. 2:**
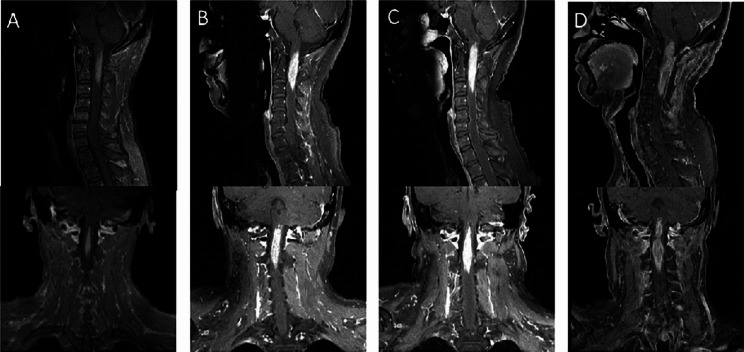
Serial coronal and sagittal postcontrast MR images at presentation and follow-up. Coronal and sagittal contrast-enhanced MRI **(A)** revealed patchy enhancement in the medulla oblongata and cervical spinal cord at presentation. Repeated enhanced-MRI after 1.5 months **(B)** showed an enlarged enhanced lesion. MRI at 2 months from presentation **(C)** showed a new enlargement of enhanced lesion. MRI at 3 months from presentation **(D)** revealed a larger lesion

**Fig. 3:**
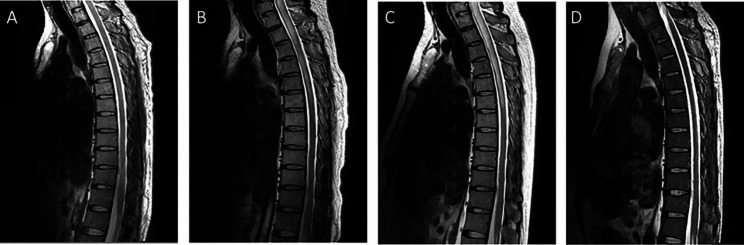
Serial sagittal MR images at presentation and follow-up. Initial sagittal image **(A)** revealed no obvious abnormality in the thoracic spinal cord. Repeated MRI after 1.5 months **(B)** showed the swelling of the thoracic cord and cord-like abnormal T2 signal. MRI at 2 months from presentation **(C)** showed similar lesion. MRI at 6 months from presentation **(D)** revealed the lesion significantly reduced

A 49-year-old man was admitted to our hospital presented with 1 month of limb numbness and developed pinprick-like pain in the posterior neck and unsteady gaits 7 days ago. Neurological examination on admission revealed brisk deep tendon reflexes and positive Babinski’s sign. Results of the rest of the neurological examination were normal. He had no underlying health problems other than type 2 diabetes. MRI of the cervical spine showed an abnormal signal with aberrant reinforcement at medulla oblongata and the level of C1-C7 (Figs. 1A and 2A). No obvious abnormalities were found on thoracic spine and optic nerves (Fig. 3A). CSF examination revealed total protein 664 mg/L (normal value 150–450), albumin 394 mg/L (normal value 100–300), lactate dehydrogenase (LDH) 25 U/L (normal value <40), IgG 64.2 mg/L (normal value ≤ 58.6), IgA 8.2 mg/L (normal value ≤ 7.0). The autoantibodies associated with paraneoplastic neurological syndrome were not detected. Tumor markers detection showed Cytokeratin fragment 19 was slightly high. Therefore, he was clinically diagnosed as longitudinally extensive transverse myelitis (LETM) (antibody-negative). Steroid pulse therapy (methylprednisolone, 1 g starting, halving every 3 days, 120 ending) was administered after which patient improved and was discharged on oral prednisolone for maintenance (Fig. 1B).

One month later, he developed limb weakness, inability to walk, severe pain in the posterior occiput, back, and waist, incontinence of urine and feces, accompanied with fever. Neurological examination revealed decreased muscle strength, increased muscle tone, hypesthesia and ataxia. MRI showed a new lesion in the thoracic myelon (Figs. 1C, 2B and 3B). We launched a new cascade of Steroid pulse therapy (methylprednisolone, 500 mg starting, halving every 3 days, 40 mg ending). But it did not improve his symptoms, which was confirmed objectively by MRI (Figs. 1D, 2C and 3C).

**Fig. 4:**
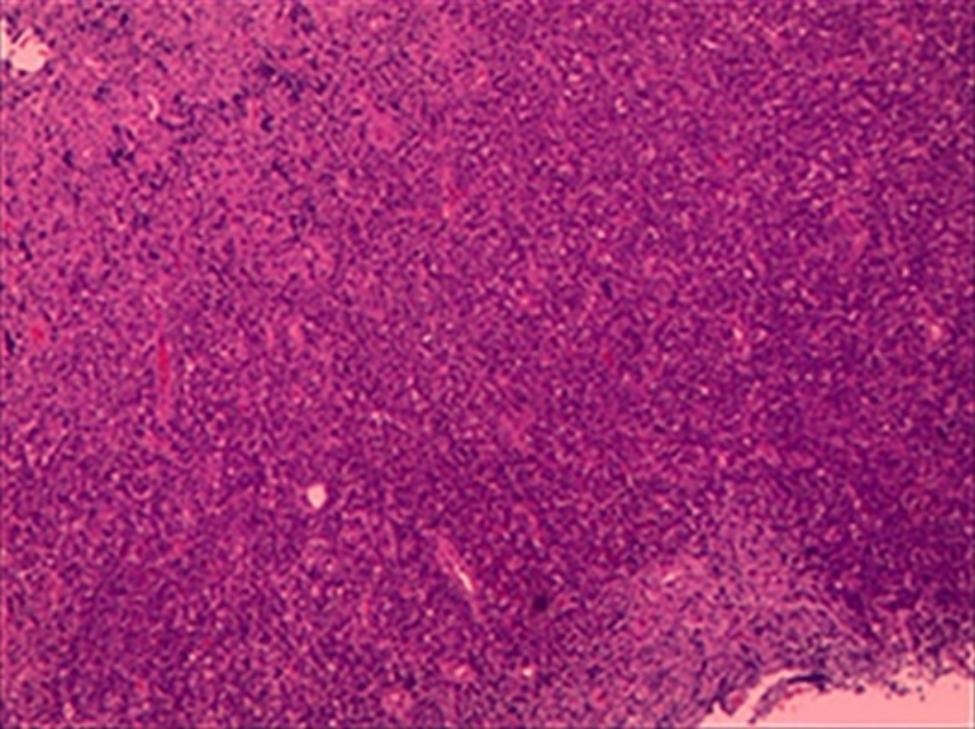
The pathological morphology observed under microscope. (hematoxylin and eosin staining, 40×)

**Fig. 5:**
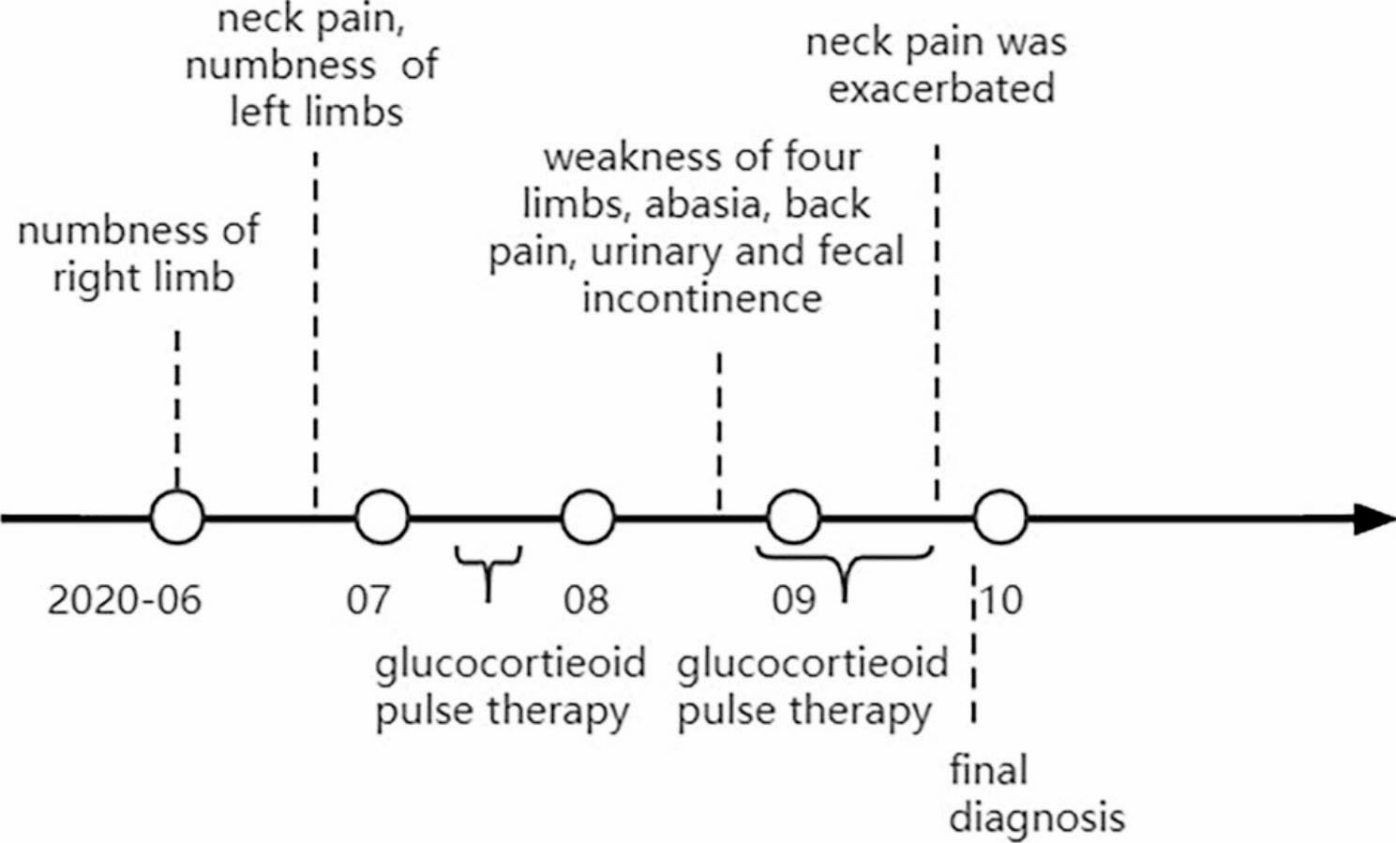
Schematic of the timeline of disease progression

After consultation with radiology and neurosurgery neoplastic lesions like glioma were considered. Vertebral microscopic resection and the postoperative pathology manifested aggressive non-Hodgkin lymphoma (non-germinal center), corresponding with diffuse large B-cell lymphoma (DLBCL). Immunohistochemistry (IHC) was positive for Lymphocyte common antigen (LCA) and cluster differentiation (CD) 20 (Fig. 4). Positron Emission Computed Tomography (PET/CT) showed hypermetabolic lesions from medulla oblongata to the cervical cord, ruling out the existence of systemic diseases. The final diagnosis is PISCL (3 months post illness onset). His disease process is listed as Fig. 5.

Then he was treated with combination chemotherapy regimens including 8 cycles of high-dose methotrexate (HD-MTX, 3.5 g/m^2^) and rituximab (RTN, 375 mg/m^2^)between October 20, 2020, and April 9, 2021. The patient breathed a sigh of relief as indicated by the MRI (Figs. 1E and F, 2D and 3D). The efficacy was evaluated as partial remission in March 2021. Next he underwent radiation therapy in the tumor area between May 2021 and June 2021. The follow-up MRI showed changes after treatment with no relapses, after which he just received palliative care. Unfortunately, pulmonary, abdomen and lymph nodes metastases were detected when he was readmitted to the hospital because of edema of both lower limbs in March 2023. He was treated with 2 cycles chemotherapy (cyclophosphamide 1000 mg + vindesine 3 mg + etoposide 0.12 g + prednisone 100 mg), resulting in very little success. At this time, he was bedridden with upper extremity muscle strength level 5- and lower extremity muscle strength level 0. Eventually, he accepted zanubrutinib for targeted therapy and a longer follow-up period was required to fully determine its effectiveness.

## Discussion and conclusions

This patient initially presented with myelopathy without any proof of antibody for demyelination and paraneoplastic syndromes, so he was misdiagnosed as LETM [3, 8]. The efficacy of the methylprednisolone therapy was maintained only for a short period, transiently improving her symptoms and reducing the size of the lesion. The long term follow-up revealed that he had a relapse after a short remission with pathogenetic condition progressively aggravated and lesions in the medulla oblongata and the cervical spinal cord extended to the thoracic spinal cord with a persistent enhancement. The CSF examination found no special positive outcomes, just revealing some outliers in routine, biochemical and immune indicators. The previous case studies also recorded the most common finding was the increased protein in CSF [9]. Multiple CSF examination may find abnormal lymphocyte populations and yield positive result, but repeat lumbar puncture is not quite applicable and tissue biopsy is still required to establish an accurate histological diagnosis [3, 10]. Consequently, there are indicators to perform the biopsy when the neurological symptoms aggravate progressively with no specific reason [11]. In this case, diagnosis is confirmed by biopsy through multidisciplinary efforts three months after the onset of illness, which won over a certain amount of time for his therapy. Most cases showed rapid disease progression within the first year after disease onset, so it is of great importance to assay Timing of biopsy [7].

Given the fact that it could be easily misdiagnosed, we encompassed a thorough search of the electronic databases of PubMed with the key terms of primary intramedullary spinal cord lymphoma in this literature review to summarize the clinical features of PISCL. We performed the initial search and selected relevant studies following title, abstract, and full-text screening. Overall, including this case, 30 studies [1, 2, 4, 9, 10, 12–35] reporting on 32 patients were incorporated in this review.

As the Table 1 shows, patients were predominantly middle-aged to elderly (average 51.9 years, median 50.5 years, range 11–82), most are in their 40 and 50 s, and more than three-fifths were women. Four of these cases made clear reference to autoimmunity or immunosuppression, corresponding with the risk factors of PCNSL [19, 36]. 94% of patients had limb weakness and 68% of patients had limb numbness. Nearly half of patients felt pain in their neck or back (44%), while very little (3%) in limbs. As the disease progresses, half of patients occurred in fecal and urinary incontinence.

**Table 1:** Literature Review: Clinical characteristics of primary intramedullary spinal cord lymphoma

Characteristic	Ratio (%)
Demographics	
Sex	
Male	13/32 (41)
Female	19/32 (59)
Age	
≥ 40	25/32 (78)
≥ 50	18/32 (56)
Clinical symptoms	
Limb weakness	29/31 (94)
Limb numbness	21/31 (68)
Neck or back pain	14/31 (45)
Limb pain	1/31 (3)
Bowel and bladder dysfunction	15/31 (48)
Ataxia	6/31 (19)
Ocular signs	4/31(13)
Distribution of lesions	
Cervical segments	17/28 (59)
Thoracic segments	19/28 (68)
Lumbar segments	8/28 (29)
Sacral segment	3/28 (11)
Extramedullary involvement	12/32 (38)

The lesion occurred more frequently in the cervical cord (59%) and thoracolumbar cord (68%), which was consistent to the previous study [29]. There were 38% of patients combining with extramedullary damages, but symptom-related data had been documented less frequently, making it hard to distinguish whether due to data collection and record or to a mismatch between brain damage and clinical manifestation.

In all series reviewed, the CSF often showed elevated CSF protein, which contributed little to final diagnosis. The most common MRI findings were the swelling spinal cord, high signals on T2-weighted images and persistent enhancement in lymphoma lesions [3, 26, 37]. Moreover, the abnormal intense high metabolic region in (18) F-fluorodeoxyglucose-positron emission tomography (FDG-PET) may help to discriminate between non‐neoplastic and neoplastic colorectal lesions [38].

The surgery, radiotherapy and chemotherapy used alone or in combination, were commonly employed for against PISCL. (Table 2) Among them, a combination of radiotherapy and chemotherapy was the most widely used therapy, accounting for exactly half of all therapeutic method. The unique advantage of surgery lay in the immediate symptom remission in the postoperative period. With regard to long-term effects, chemotherapy separately was more beneficial compared with other therapy methods [1, 5, 38]. The dose of radiotherapy was connected with the efficacy and the patients might be at great risk of poor response to treatment and recurrence when the radiation dose was less than 40 Gy [39]. Hormone therapy was effective, but it was short in maintaining remission [40].

**Table 2:** Summary of reported cases of primary intramedullary spinal cord lymphoma

Entry	Ratio (%)
Pathologic type	
B cell	12/17 (71)
T cell	5/17 (29)
Treatment	
Surgery, chemotherapy and radiation	3/20 (15)
Surgery and chemotherapy	1/20 (5)
Surgery and radiation	2/20 (10)
Chemotherapy and radiation	10/20 (50)
Chemotherapy	2/20 (10)
Radiation	1/20 (5)
Surgery Prognosis	1/20 (5)
1-year survival	9/12 (75)
2-year survival	5/12 (42)
3-year survival	3/12 (25)
4-year survival	3/12 (25)

Demographic variables (age, race, year of diagnosis, marital status) and tumor characteristics (anatomic location, stage and histology) were related to patient prognosis [7, 39]. The disease progressed rapidly during the first two years after onset and then tended to be flat (Table 1). Therefore, early and rapid diagnosis, timely and appropriate treatment of PISCL are extremely crucial in improving the survival of the patients.

Most PISCL patients are presumed to be demyelinating diseases of the central nervous system initially because of similar clinical manifestations and conventional MRI features and eventually confirmed through biopsy or autopsy. The misdiagnosis of the present case is due to the biopsy was not taken at the time of first diagnosis. But because it is invasive, it is not always applied for assessment of inflammatory lesions. Thus, the timing of biopsy is very important. A biopsy is recommended when the high-risk groups such as older age or immune deficiency [7, 19, 36, 41, 42] not clinically good after processed as inflammation.

These observations emphasized the need to develop some reliable biomarkers to avoid diagnosis delays. There are reports in the literature claiming that serum miR-21 and neopterin, myeloid differentiation primary response (88) (MYD88) L265P mutation (mut-MYD88) and interleukin-10 (IL-10) in CSF seem to be promising biomarkers in the diagnosis of PCNSL [43–45]. Further research is needed to confirm and extend current findings.

It is worth noting that inflammatory changes may be a prodrome of PCNSL [46]. Some patients did not find typical lymphocytic infiltration but inflammatory changes in their first biopsy and got a positive result after repeat biopsy, which may propose a new challenge in diagnosis. Nevertheless, it may be helpful to unravel the pathogenesis of PCNSL.

At the same time, the recurring problem should be taken into consideration. Patients were advised to receive follow-ups and clinical reassess at regular intervals after therapy, especially within 2 years of initial therapy [47], checking whether there were local-regional recurrence or even distant recurrence.

This case illustrates the whole process from misdiagnosis to correct diagnosis in detail, demonstrating the natural course of PISCL in a way. We must recognize that PISCL, while rare, often poses a diagnostic challenge. In case of spinal onset patients react ineffectively to standard immunoglobulins or hormonal treatments or experience a relapse after a short time relief, it is necessary to perform not only a biopsy, but also an optimally possible decompression. Early diagnosis is crucial to therapeutic efficacy.

## Data Availability

Data sharing is not applicable to this article as no datasets were generated or analysed during the current study.

## Abbreviations

PISCL: Primary intramedullary spinal cord lymphoma
LETM: Longitudinally extensive transverse myelitis
DLBCL: Diffuse large B-cell lymphoma
